# Tracking the mental health of a nation: prevalence and correlates of mental disorders in the second Singapore mental health study

**DOI:** 10.1017/S2045796019000179

**Published:** 2019-04-05

**Authors:** M. Subramaniam, E. Abdin, J. A. Vaingankar, S. Shafie, B. Y. Chua, R. Sambasivam, Y. J. Zhang, S. Shahwan, S. Chang, H. C. Chua, S. Verma, L. James, K. W. Kwok, D. Heng, S. A. Chong

**Affiliations:** 1 Research Division, Institute of Mental Health, Singapore; 2 Lee Kong Chian Medical School, Singapore; 3 Department of General Psychiatry, Institute of Mental Health, Singapore; 4 Clinical Education, Office of Education, Duke-NUS Medical School, Singapore; 5 Department of Psychosis and East Region, Institute of Mental Health, Singapore; 6 Epidemiology & Disease Control Division, Ministry of Health, Singapore; 7 President's Office, Nanyang Technological University, Singapore

**Keywords:** Composite International Diagnostic Interview, multi-ethnic, prevalence, Singapore, survey

## Abstract

**Aims:**

The second Singapore Mental Health Study (SMHS) – a nationwide, cross-sectional, epidemiological survey - was initiated in 2016 with the intent of tracking the state of mental health of the general population in Singapore. The study employed the same methodology as the first survey initiated in 2010. The SMHS 2016 aimed to (i) establish the 12-month and lifetime prevalence and correlates of major depressive disorder (MDD), dysthymia, bipolar disorder, generalised anxiety disorder (GAD), obsessive compulsive disorder (OCD) and alcohol use disorder (AUD) (which included alcohol abuse and dependence) and (ii) compare the prevalence of these disorders with reference to data from the SMHS 2010.

**Methods:**

Door-to-door household surveys were conducted with adult Singapore residents aged 18 years and above from 2016 to 2018 (*n* = 6126) which yielded a response rate of 69.0%. The subjects were randomly selected using a disproportionate stratified sampling method and assessed using World Health Organization Composite International Diagnostic Interview version 3.0 (WHO-CIDI 3.0). The diagnoses of lifetime and 12-month selected mental disorders including MDD, dysthymia, bipolar disorder, GAD, OCD, and AUD (alcohol abuse and alcohol dependence), were based on the Diagnostic and Statistical Manual of Mental Disorders, Fourth Edition (DSM-IV) criteria.

**Results:**

The lifetime prevalence of at least one mood, anxiety or alcohol use disorder was 13.9% in the adult population. MDD had the highest lifetime prevalence (6.3%) followed by alcohol abuse (4.1%). The 12-month prevalence of any DSM-IV mental disorders was 6.5%. OCD had the highest 12-month prevalence (2.9%) followed by MDD (2.3%). Lifetime and 12-month prevalence of mental disorders assessed in SMHS 2016 (13.8% and 6.4%) was significantly higher than that in SMHS 2010 (12.0% and 4.4%). A significant increase was observed in the prevalence of lifetime GAD (0.9% to 1.6%) and alcohol abuse (3.1% to 4.1%). The 12-month prevalence of GAD (0.8% vs. 0.4%) and OCD (2.9% vs. 1.1%) was significantly higher in SMHS 2016 as compared to SMHS 2010.

**Conclusions:**

The high prevalence of OCD and the increase across the two surveys needs to be tackled at a population level both in terms of creating awareness of the disorder and the need for early treatment. Youth emerge as a vulnerable group who are more likely to be associated with mental disorders and thus targeted interventions in this group with a focus on youth friendly and accessible care centres may lead to earlier detection and treatment of mental disorders.

## Introduction

Mental disorders are common and pervasive. Recent systematic reviews have reported high prevalence of mental disorders worldwide, but a comparatively low prevalence in East and Southeast Asia even after adjusting for methodological differences (Ferrari *et al*., 2013; Steel *et al*., 2014). According to a recent WHO report, the proportion of the global population with depression in 2015 was estimated to be 4.4% while those with anxiety disorders was 3.6%. The total estimated number of people living with depressive and anxiety disorders had increased from 2005 to 2015, which is attributable to the overall growth of the global population, ageing, as well as a proportionate increase in the age groups at which depression is more prevalent (WHO, 2017).

The first Singapore Mental Health Study was initiated in 2010 (Subramaniam *et al*., 2012*a*) (hereafter referred to as SMHS 2010). This nationwide epidemiological study found that the prevalence of at least one life-time mood, anxiety, or AUD was 12.0% in the adult population of Singapore (Chong *et al*., 2012). While this study provided important data pertaining to the mental health of the nation, the findings also serve as a baseline to track the trend of the subsequent mental health status of the general population as well as to evaluate the effectiveness of population-wide interventions/ programmes that had been initiated and implemented in the intervening years.

Epidemiological surveys have compared the prevalence of mental disorders across different countries (Bromet *et al*., 2011; Corbani *et al*., 2017) but only a few studies have made comparisons over time within countries using the same methodology. Repeated surveys conducted across time in Canada (Patten *et al*., 2016), Korea (Cho *et al*., 2015) and Japan (Ishikawa *et al*., 2018) have reported differing findings. Patten *et al*. (2016) found that both the lifetime and 12-month prevalence of major depressive episodes did not increase from 2002 to 2012 in Canada, while Cho *et al*. (2015) reported that the 12-month prevalence of MDD and anxiety disorders increased from 2006 to 2011, while the prevalence of nicotine dependence and AUD decreased in the same period in Korea. Ishikawa *et al*. (2018) found that the 12-month prevalence of mood and substance use disorders were slightly higher in the second World Mental Health Japan Survey conducted in 2013–2015) (WMHJ2) as compared to the first World Mental Health Japan Survey conducted in 2002–2006 (WMHJ1) while the prevalence of GAD and post-traumatic stress disorder were significantly lower in the WMHJ2 as compared to WMHJ1. These results suggest that prevalence of mental disorders changes across time and that these changes may be specific to countries/regions.

The second SMHS was carried out in 2016 (hereafter referred to as SMHS 2016) with the intent of tracking the state of mental health of Singapore. The two studies (SMHS 2016 and SMHS 2010) employed the same methodology i.e., similar sampling strategy, same instrument – WMH CIDI 3.0, which was administered using computer assisted personal interviewing (CAPI), and the same trainers were involved in the training of the interviewers and quality assessments of interviews and data using standardised protocols.

The SMHS 2016 aimed to (i) establish the 12-month and lifetime prevalence and correlates of MDD, dysthymia, bipolar disorder, GAD, OCD and AUD (which included alcohol abuse and dependence) and (ii) compare the prevalence of these disorders with reference to data from the SMHS 2010.

## Materials and methods

### Survey population and subjects

The SMHS 2016 was conducted following the same procedures as the SMHS 2010 (Subramaniam *et al*., 2012*a*). This population-based, cross-sectional epidemiological study included Singapore residents aged 18 years and above living in Singapore. The sampling frame was based on a national population registry database of all citizens and permanent residents in Singapore which is updated regularly. A probability sample was randomly selected using a disproportionate stratified sampling design with 16 strata defined according to ethnicity (Chinese, Malay, Indian, Others) and age groups (18–34, 35–49, 50–64, 65 and above). Residents aged 65 and above, Malays and Indians were over-sampled to ensure that sufficient sample size would be achieved to improve the reliability of estimates for the subgroup analysis. The sampling frames used in 2010 and 2016 were independent of each other, since the data is completely de-identified at the completion of the survey, we were unable to determine if some of the participants were common to both surveys.

The sample size numbers were produced by running statistical power calculations for binary proportions to determine what sample sizes are necessary overall, as well as for sub-groups, to produce a precise estimate with a margin of error equal to 0.05 for different disorders. A statistical power of 0.80 was assumed, while the Type 1 error rate is controlled at *α* = 0.05, as is standard. Note that if Type 2 error = ß, then power = 1- ß. Power calculations for estimated rates of mental disorders were generated from low prevalence (GAD = 0.9%) to high prevalence disorders (MDD = 5.8%) as identified in SMHS 2010 (Chong *et al*., 2012). The sample size was adjusted to account for deviations from simple random sampling. The estimated design effect (DEFF) after oversampling on age (those aged >65) and ethnicity (Malay and Indian) was 1.942. Realistic sample sizes were assumed (e.g., *n* = 5,500, 6000 and 6500) and margin of error was then computed for each disorder. We found the target sample size of 6000 adequate to provide sufficient precision to measure the prevalence of all disorders. The margin of error for the overall prevalence estimate was between 0.3% to 0.8%, while the margin of error for the subgroups defined by age and ethnic groups was between 0.7% to 1.8%.

A total number of 6126 respondents were interviewed. The response rate among the eligible adults was 69.0% and the acceptability of the interview was high as there were very few who agreed to begin the interview but did not complete it subsequently (incomplete cases = 25).

### Study procedures

An invitation letter was sent to each subject followed by a personal home visit by a trained interviewer to obtain his/her agreement to participate in the survey. Trained interviewers from a survey research company conducted face-to-face interviews with those who agreed to participate in the study. The questionnaires were available in English, Chinese and Malay and respondents were asked to choose the language they were most comfortable with before the interviewer initiated any study related procedures. Those residents who were incapable of doing an interview due to severe physical or mental conditions, language barriers, were living outside the country, institutionalized or hospitalized at the time of the survey, and those who were not contactable due to incomplete or incorrect address, were excluded from the survey.

### Questionnaires

#### WHO-CIDI

The fully-structured CAPI version of the WHO-CIDI 3.0 (Kessler and Ustün, 2004) was used in the study. The CIDI 3.0 assesses lifetime and 12-month prevalence of disorders using the definitions and criteria of the DSM-IV (APA, 1994) as well as the International Classification of Disease, 10th Revision (ICD-10) Classification of Mental and Behavioral Disorders (WHO, 1992). A screening section was administered to all respondents. All participants who answered positively to a specific screening question were then referred to the respective diagnostic section of the questionnaire.

Only select modules of the CIDI were included in the survey. These modules were identified based on inputs from a Stakeholder Board comprising representatives from various stakeholders (Ministry of Health, voluntary organizations working with mentally ill clients, clinicians, sociologists, and representatives from the major ethnic groups in Singapore) during SMHS 2010 and the decision to include them was revisited with the policy makers before the current survey. The disorders were selected based on the relevance to the country and its policy-makers and service providers. This approach was also taken to alleviate respondent burden which coupled with the extant stigma towards mental illnesses in Asian populations (Subramaniam *et al*., 2017*a*) could affect the overall response rate. All diagnoses were made using organic exclusions and diagnostic hierarchy rules.

#### Socio-demographic questionnaire

Data on sex, age, ethnicity (Chinese, Malay, Indian, and Others), marital status (single, married, divorced/separated or widowed), educational level (primary and below, secondary, pre-university/junior college, vocational/ITE, diploma and university), employment status (employed, unemployed and economically inactive i.e., students, homemakers and retirees) and household income was collected. Household income was calculated as the sum of all pre-tax income in the past 12 months, of all family members living in the same household.

#### Statistical analysis

In order to account for the stratified disproportionate sampling design and ensure that the survey findings were representative of the Singapore adult population, all estimates were weighted to adjust for over sampling, non-response and post-stratified for age and ethnicity distributions between the survey sample and the Singapore resident population in 2014. Descriptive analyses were performed to establish 12-month and lifetime prevalence of mental disorders as well as describe the socio-demographic profile of the study population. Persistence of disorders was estimated with two prevalence ratios; the 12-month prevalence among lifetime cases and 30-day prevalence among 12-month cases (Kessler *et al*., 2012). Associations between lifetime mental disorders and socio-demographic variables were examined using logistic regression. To examine the change in prevalence across the two surveys, data from SMHS-2016 were reanalyzed using similar sampling weight as the SMHS-2010 study. Significant differences in prevalence were tested using Chi-square tests followed by pooled logistic-regression analyses (Kessler *et al*., 2005) with time (0 = year 2010, 1 = year 2016) treated as a primary predictor of each disorder after controlling for age, gender and ethnicity. Standard errors (SE) and significance tests were estimated using the Taylor series linearization method. All statistical analyses were performed using the Statistical Analysis Software (SAS) system version 9.3 (Cary, NC, USA).

## Results

### Socio-demographic characteristics of the sample

Supplementary Table 1 shows the socio-demographic distribution of the sample.

### Lifetime and 12-month prevalence and persistence of disorders

Table 1 shows the weighted prevalence of lifetime and 12-month mental disorders in the population. Lifetime prevalence of at least one mood, anxiety or alcohol use disorder was 13.9% (95% CI: 12.7–15.2) in the adult population. The lifetime prevalence of mood disorders was 8.0% (95% CI: 7.0–9.0) while that of anxiety disorders (GAD and OCD only) was 4.8% (95% CI: 4.1–5.6). The prevalence of AUD was 4.7% (95% CI: 3.9–5.5). The 12-month prevalence of any DSM-IV mental disorder was 6.5% (95% CI: 5.7–7.4) and anxiety disorders had the highest 12-month prevalence (3.6%, 95% CI: 3.0–4.3), followed by mood disorders (3.3%, 95% CI: 2.7–4.0).

**Table 1.** Lifetime and 12-month Weighted Prevalence of Mental Disorders in the Singapore Mental Health Study 2016

Disorder	Lifetime Prevalence %	12-Month Prevalence %	30-day Prevalence %	12-month/Lifetime Prevalence ratio	30-day/12-Month Prevalence ratio
Major Depressive Disorder	6.3	2.3	0.4	37.1	19.1
Dysthymia	0.3	0.2	0.1	75.5	48.8
Bipolar Disorder	1.6	0.9	0.3	55.4	32.5
Generalized Anxiety Disorder	1.6	0.8	0.2	53.3	18.6
Obsessive Compulsive Disorder	3.6	2.9	1.7	81.5	59.9
Alcohol Abuse	4.1	0.6	0.1	13.6	23.7
Alcohol Dependence	0.5	0.2	0.1	41.1	37.2
Any Mental Disorder	13.9	6.5	2.7	46.7	58.8
Two or more mental disorder	3.5	1.3	0.3	37.2	19.9

Any mental disorder: Has at least one of the mental disorders assessed by the Composite International Diagnostic Interview in the study.

Table 2 shows the distribution of the lifetime prevalence of mental disorders (as diagnosed by DSM-IV criteria) by age, gender and ethnic status. Table 3 shows the distribution of the 12-month prevalence of mental disorders (as diagnosed by DSM-IV criteria) by age, gender and ethnic status.

**Table 2.** Prevalence of lifetime mental disorders by age, gender and ethnicity in the Singapore Mental Health Study 2016

	MDD	Dysthymia	Bipolar	GAD	OCD	Alcohol abuse	Alcohol Dependence	Two or more mental disorders	Any Mental Disorder
**Age groups (years)**									
18–34	9.2	0.7	2.8	2.2	6.7	6.3	0.9	6.2	21.6
35–49	7.7	0.2	1.5	1.9	3.9	5.0	0.5	4.5	15.4
50–64	3.1	0.0	0.9	1.0	1.0	2.3	0.2	0.7	7.9
65 +	2.7	0.2	0.1	0.6	0.9	0.9	0.2	0.6	5.0
**Gender**									
Male	5.6	0.2	1.5	1.6	3.1	6.6	0.9	3.7	15.0
Female	7.0	0.4	1.6	1.6	4.1	1.7	0.1	3.3	12.8
**Ethnicity**									
Chinese	6.4	0.2	1.4	1.5	3.4	4.0	0.4	3.4	13.4
Malay	4.9	0.4	2.0	1.7	5.4	3.4	1.0	3.6	14.5
Indian	7.5	1.0	2.4	2.0	3.1	5.2	1.1	4.4	17.0
Others	6.0	−	1.9	2.4	3.0	6.0	0.4	3.0	16.4

Any mental disorder: Has at least one of the mental disorders assessed by the Composite International Diagnostic Interview in the study; GAD Generalised anxiety disorder; MDD Major Depressive Disorder; OCD Obsessive Compulsive Disorder.

**Table 3.** Prevalence of 12-month mental disorders by age, gender and ethnicity in the Singapore Mental Health Study 2016

	MDD	Dysthymia	Bipolar	GAD	OCD	Alcohol abuse	Alcohol dependence	Two or more mental disorders’	Any mental disorder
**Age groups (years)**									
18–34	4.4	0.5	2.1	1.1	5.7	1.1	0.4	3.1	11.9
35–49	1.9	0.1	0.4	1.3	3.1	0.5	0.2	1.0	6.3
50–64	1.3	0.03	0.4	0.5	0.8	0.2	0.03	0.1	3.1
65 +	0.5	0.2	0.03	0.04	0.5	0.02	0.2	0.2	1.3
**Gender**									
Male	2.3	0.2	0.9	0.9	2.1	0.9	0.4	1.2	6.4
Female	2.4	0.3	0.8	0.8	3.7	0.2	0.1	1.4	6.6
**Ethnicity**									
Chinese	2.2	0.2	0.7	0.8	2.8	0.5	0.2	1.2	6.0
Malay	2.9	0.3	1.2	0.8	4.3	0.3	0.3	1.7	8.2
Indian	3.0	0.8	1.7	1.2	2.2	1.0	0.5	1.9	8.0
Others	1.7	.	1.2	0.8	2.9	0.9	0.4	0.8	7.0

Any mental disorder: Has at least one of the mental disorders assessed by the Composite International Diagnostic Interview in the study; GAD Generalised anxiety disorder; MDD Major Depressive Disorder; OCD Obsessive Compulsive Disorder.

### Socio-demographic correlates of lifetime mental disorders

The results of logistic regression analyses examining the association of socio-demographic predictors with lifetime prevalence, of DSM-IV disorders are shown in Table 4.

**Table 4.** Sociodemographic correlates for lifetime mental disorders in the Singapore Mental Health Study 2016

	MDD				Bipolar				GAD				OCD			
	OR	95% CI		*p* value	OR	95% CI		*p* value	OR	95% CI		*p* value	OR	95% CI		*p* value
**Age group**																
18–34 (reference)																
35–49	0.8	0.5	1.3	0.419	0.4	0.2	0.8	0.013	1.4	0.5	3.8	0.563	0.6	0.3	1.0	0.064
50–64	0.3	0.2	0.6	<0.001	0.3	0.1	0.8	0.023	1.0	0.3	3.5	0.968	0.1	0.1	0.2	<0.001
65 +	0.3	0.1	0.7	0.004	0.1	0.0	0.2	<0.001	0.6	0.2	2.3	0.439	0.1	0.0	0.3	<0.001
**Gender**																
Male (reference)																
Female	1.3	0.9	1.8	0.165	1.3	0.7	2.4	0.449	0.9	0.5	1.8	0.849	1.5	1.0	2.2	0.084
**Ethnicity**																
Chinese (reference)																
Malay	0.8	0.6	1.2	0.309	0.9	0.5	1.6	0.639	1.2	0.6	2.3	0.630	1.6	1.0	2.4	0.030
Indian	1.2	0.9	1.6	0.226	1.3	0.7	2.3	0.405	1.4	0.8	2.4	0.292	0.9	0.6	1.3	0.466
Others	0.9	0.5	1.4	0.625	1.5	0.6	3.5	0.370	1.7	0.7	3.9	0.214	0.8	0.4	1.6	0.553
**Marital**																
Married (reference)																
Never married	1.2	0.8	1.9	0.439	0.9	0.5	1.7	0.738	2.4	0.9	6.5	0.081	1.2	0.7	2.1	0.547
Divorced/separated	3.9	2.2	7.1	<0.001	3.0	1.1	8.4	0.034	2.7	0.9	8.4	0.089	1.4	0.5	3.9	0.577
Widowed	1.8	0.5	5.9	0.359	0.1	0.0	1.1	0.058	3.0	0.7	12.4	0.128	0.3	0.1	1.3	0.108
**Education**																
University (reference)																
Primary and below	0.8	0.4	1.8	0.633	0.2	0.0	0.8	0.019	0.2	0.1	0.7	0.012	1.3	0.5	3.2	0.539
Secondary	0.9	0.5	1.5	0.707	1.5	0.5	4.5	0.491	0.6	0.2	1.5	0.247	0.9	0.5	1.8	0.801
Pre-U/Junior College	1.5	0.7	3.0	0.257	1.0	0.1	6.6	0.980	0.6	0.1	3.0	0.567	1.2	0.5	3.2	0.680
Vocational/ITE	1.0	0.5	2.2	0.902	1.2	0.5	2.9	0.675	1.2	0.4	3.6	0.740	0.9	0.4	2.0	0.804
Diploma	1.1	0.7	1.7	0.790	1.5	0.6	3.7	0.361	0.9	0.3	2.2	0.767	1.1	0.6	2.0	0.854
**Employment**																
Employed (reference)																
Economically inactive	0.8	0.5	1.4	0.473	0.3	0.1	0.9	0.040	0.6	0.3	1.3	0.189	0.6	0.4	1.1	0.128
Unemployed	1.3	0.7	2.5	0.456	3.1	1.2	8.2	0.022	1.6	0.5	4.9	0.433	1.4	0.7	2.9	0.374
**Income (SGD/ month)**																
Below 2000 (reference)																
2000–3999	0.6	0.3	1.0	0.062	1.2	0.5	3.2	0.692	0.7	0.2	2.0	0.525	0.5	0.3	0.9	0.027
4000–5999	0.6	0.3	1.1	0.078	0.7	0.2	2.2	0.547	1.1	0.4	3.2	0.810	0.6	0.3	1.1	0.091
6000–9999	0.9	0.5	1.6	0.714	0.5	0.2	1.8	0.313	1.0	0.3	3.0	0.934	0.6	0.3	1.3	0.236
10 000 & above	1.0	0.5	2.0	0.891	0.3	0.1	1.2	0.099	0.6	0.1	2.2	0.406	0.6	0.3	1.3	0.201

	Alcohol abuse				Two or more mental disorders				Any mental disorder			
	OR	95% CI		*p* value	OR	95% CI		*p* value	OR	95% CI		*p* value
**Age group**												
18–34 (reference)												
35–49	0.7	0.4	1.3	0.221	0.9	0.5	1.6	0.700	0.6	0.4	0.8	0.003
50–64	0.2	0.1	0.5	<0.001	0.1	0.04	0.2	<0.001	0.3	0.2	0.4	<0.001
65 +	0.1	0.0	0.2	<0.001	0.1	0.03	0.3	<0.001	0.2	0.1	0.3	<0.001
**Gender**												
Male (reference)												
Female	0.3	0.2	0.5	<0.001	0.9	0.5	1.3	0.499	0.9	0.7	1.1	0.377
**Ethnicity**												
Chinese (reference)												
Malay	0.6	0.4	0.9	0.022	0.9	0.6	1.4	0.655	1.0	0.8	1.3	0.962
Indian	1.1	0.7	1.6	0.704	1.2	0.8	1.8	0.328	1.3	1.0	1.6	0.041
Others	1.8	1.1	3.1	0.029	0.9	0.5	1.8	0.834	1.3	0.9	1.7	0.156
**Marital**												
Married (reference)												
Never married	0.9	0.5	1.7	0.725	1.9	1.1	3.6	0.029	1.0	0.7	1.4	0.973
Divorced/separated	1.7	0.7	4.1	0.199	3.6	1.6	8.3	0.003	2.7	1.7	4.3	<0.001
Widowed	0.9	0.2	5.0	0.882	1.3	0.3	6.0	0.756	1.0	0.4	2.5	0.964
**Education**												
University (reference)												
Primary and below	3.6	1.5	9.0	0.005	2.3	0.9	5.6	0.055	1.0	0.6	1.7	0.997
Secondary	2.3	1.2	4.7	0.018	1.2	0.6	2.7	0.581	1.1	0.8	1.7	0.492
Pre-U/Junior College	0.6	0.1	3.0	0.516	1.2	0.4	3.7	0.795	1.1	0.6	2.0	0.647
Vocational/ITE	2.9	1.4	6.0	0.005	0.9	0.4	2.0	0.758	1.6	1.0	2.4	0.053
Diploma	1.6	0.9	3.1	0.116	1.7	0.9	3.1	0.112	1.0	0.7	1.5	0.797
**Employment**												
Employed (reference)												
Economically inactive	0.4	0.2	1.0	0.048	0.5	0.2	0.9	0.034	0.6	0.4	0.9	0.011
Unemployed	1.3	0.6	2.9	0.508	1.1	0.5	2.5	0.871	1.7	1.1	2.7	0.019
**Income (SGD/ month)**												
Below 2000 (reference)												
2000–3999	0.8	0.4	1.4	0.403	0.5	0.3	1.01	0.054	0.7	0.5	1.1	0.105
4000–5999	0.4	0.2	0.8	0.010	0.5	0.2	0.9	0.033	0.6	0.4	0.9	0.019
6000–9999	0.8	0.4	1.6	0.460	0.5	0.2	0.9	0.047	0.9	0.6	1.4	0.735
10 000 & above	1.0	0.5	2.2	0.992	0.5	0.2	1.2	0.143	0.9	0.5	1.4	0.557

Any mental disorder: Has at least one of the mental disorders assessed by the Composite International Diagnostic Interview in the study; ITE: Institute of Technical Education; Pre-U Pre-University; SGD Singapore Dollar.

Note: Due to lower number of cases, regression was not estimated for dysthymia and alcohol dependence.

The odds of MDD were lower among those aged 50–64 years (OR, 0.3; 95%CI, 0.2–0.6) and 65 years and above (OR, 0.3; 95%CI, 0.1–0.7) as compared to those aged 18–34 years. A similar pattern was observed for OCD and alcohol abuse, with lower odds of OCD and alcohol abuse among those aged 50–64 years (OR, 0.1; 95%CI, 0.1–0.2 and OR, 0.2; 95%CI, 0.1–0.5 respectively) as well as those aged 65 years and above (OR, 0.1; 95%CI, 0.01–0.3 and OR, 0.1; 95%CI, 0.01–0.2) as compared to those aged 18–34 years. Those aged 18–34 years had higher odds of bipolar disorder or any mental disorder as compared to all other age groups.

Malays had higher odds of OCD (OR, 1.6; 95%CI, 1.01–2.4) and lower odds of alcohol abuse (OR, 0.6; 95%CI, 0.4–0.9) as compared to those of Chinese ethnicity. Those who were divorced or separated were more likely to have MDD (OR, 3.9; 95%CI, 2.2–7.1), bipolar disorder (OR, 3.0; 95% CI, 1.1–8.4) and any mental disorder (OR, 2.7; 95% CI, 1.7–4.3) as compared to those who were married.

### Changes in lifetime and 12-month prevalence of mental disorders from 2010 to 2016

Lifetime prevalence of mental disorders assessed in SMHS 2016 (13.8%) was significantly higher than that in SMHS 2010 (12.0%) (*p* < 0.005) (Table 5). Lifetime prevalence of any mood disorder was not different (7.9% vs. 7.0%, *p* = 0.058), while lifetime prevalence of anxiety disorders (4.8% vs. 3.6%, *p* = 0.007) and AUD (4.6% vs. 3.6%, *p* = 0.024) were significantly higher in the SMHS 2016 than in the SMHS 2010.

**Table 5.** Lifetime prevalence of Mental Disorders in the Singapore Mental Health Study 2010 (*n* = 6616) and the Singapore Mental Health Study 2016 (*n* = 6126)

Disorder	2010	2016^a^	Risk Ratio	*p* value^b^	*p* value^c^
	%	%			
**Lifetime Prevalence**					
MDD	5.8	6.2	1.07	0.479	0.269
Dysthymia	0.3	0.3	1.10	0.824	0.736
Bipolar disorder	1.2	1.6	1.28	0.233	0.134
GAD	0.9	1.6	1.79	0 .010	0.005
OCD	3.0	3.5	1.18	0.220	0.097
Alcohol Abuse	3.1	4.1	1.30	0.048	0.030
Alcohol Dependence	0.5	0.5	1.14	0.669	0.581
Any disorder	12.0	13.8	1.15	0.033	0.007
Two or more mental disorders	2.5	3.5	1.4	0.024	0.007

a To compare the prevalence between two surveys, data from SMHS-2016 were reanalyzed using similar sampling weight as the SMHS-2010 study.

b Chi-Square Test.

c Pooled logistic-regression analyses with time (0 = year 2010, 1 = year 2016) treated as a primary predictor of each disorder after controlling for age, gender and ethnicity.

Statistical significant was evaluated at the *p* value <0.05.

12-month prevalence of any mental disorder increased significantly from SMHS 2010 (4.4%) to SMHS 2016 (6.4%) (Table 6). Prevalence of anxiety disorders was significantly higher (3.6% vs. 1.5%, *p* < 0.001) in the SMHS 2016 than in the SMHS 2010. The 12-month prevalence of GAD (0.8% vs. 0.4%, *p* = 0.033) and OCD (2.9% vs. 1.1%, *p* < 0.001) were significantly higher in SMHS 2016 as compared to SMHS 2010.

**Table 6.** 12-month prevalence of Mental Disorders in the SMHS 2010 (*n* = 6616) and the SMHS 2016 (*n* = 6126)

Disorder	2010	2016^a^	Risk Ratio	*p* value^b^	*p* value^c^
	%	%			
**12-Month Prevalence**					
MDD	2.2	2.3	1.02	0.882	0.641
Dysthymia	0.3	0.2	0.83	0.670	0.735
Bipolar disorder	0.6	0.9	1.44	0.178	0.120
GAD	0.4	0.8	1.90	0.046	0.033
OCD	1.1	2.9	2.52	<0.001	<0.001
Alcohol Abuse	0.5	0.6	1.16	0.675	0.555
Alcohol Dependence	0.3	0.2	0.73	0.500	0.570
Any disorder	4.4	6.4	1.46	0.003	<0.001
Two or more mental disorders	0.9	1.3	1.38	0.160	0.089

a To compare the prevalence between two surveys, data from SMHS-2016 were reanalyzed using similar sampling weight as the SMHS-2010 study; Statistical significant was evaluated at the *p* value <0.05.

b Chi-Square Test.

c Pooled logistic-regression analyses with time (0 = year 2010, 1 = year 2016) treated as a primary predictor of each disorder after controlling for age, gender and ethnicity.

## Discussion

This study is the second, national mental health survey using a similar methodology as the previous national study to assess and compare the prevalence of mental disorders in Singapore based on a representative sample of community residents.

The high prevalence of lifetime and 12-month OCD is somewhat unique to this population. The 12-month to lifetime persistence ratio was the highest for OCD in the current study and this with the lower 30-day to 12-month prevalence ratio suggests that the disorder is highly persistent due to episode recurrence rather than chronicity. The lifetime and 12-month prevalence of OCD were 0.7% and 0.6% respectively in the Korean population (Cho *et al*., 2015). A meta-analysis of studies from China reported the current and lifetime prevalence as 0.9% and 3.2% (Guo *et al*., 2016). The authors found that individuals in urban areas were likely to have a higher risk of OCD than those in rural areas. Singapore is a city-state-country and the urban nature may lend to stress in the form of competition, higher workload and a strive for perfectionism which may contribute to the higher risk of OCD (Wu and Cortesi, 2009; Soreni *et al*., 2014).

Except for OCD, the observed lifetime and 12-month prevalence of the other disorders were still lower than that reported from Western populations but similar to that reported from other Asian countries like Japan and Korea except for AUD which was lower in the Singapore study (Demyttenaere *et al*., 2004; Steel *et al*., 2014). The low prevalence of common mental disorders in Asian countries has been noted in previous studies (Shen *et al*., 2006; Park *et al*., 2008; Ishikawa *et al*., 2016). While it is possible that the lower prevalence is a result of under-reporting by the population due to the stigma of mental illnesses (Subramaniam *et al*., 2017*a*), the lower population risk for common mental disorders in Singapore may be due to other factors like cultural stoicism (Liao *et al*., 2012) and/or the use of DSM-IV criteria which are pre-dominantly based on affective symptoms rather than the expression of somatic symptoms in Asian populations (Ryder *et al*., 2008). Further, certain social determinants of mental health like high quality antenatal care, compulsory primary education, social support, low rates of crime/violence, low unemployment rates and attributes of built environment promoting health – facets of Singapore – may have influenced the risk of mental disorders.

Our study found a significant increase in the prevalence of both lifetime and 12-month anxiety disorders. The high persistence of OCD coupled with the wide treatment gap in the local population (Subramaniam *et al*., 2012*b*) could explain the increase in the 12-month prevalence of OCD. The increase in the prevalence of GAD is however more difficult to explain. It is possible that excessive use of smartphones and associated phenomenon like ‘nomophobia’ (Cheever *et al*., 2014) may contribute to anxiety in countries like Singapore which has one of the world's highest mobile connectivity (GSMA, 2018). A recent review by De-Sola Gutiérrez *et al*. (2016), suggests that anxiety may be related with problematic cell-phone use while problematic internet use may be more likely to be associated with depression. However, the current study did not assess the extent of internet or smartphone use in the population; given that excessive use of smartphone and the effects of its restriction are a relatively new phenomenon, more research is needed before drawing any definite conclusions.

Prevalence of lifetime AUD increased from SMHS 2010 to SMHS 2016. Globalization, economic development, adoption of a more liberal culture and normalization of alcohol use have all led to increased drinking in the Asian region (Tang *et al*., 2013; World Health Organization, Mental Health and Substance Abuse Unit, 2013). Data from WHO suggests that the per capita consumption of alcohol is increasing in China, Malaysia and Singapore (Babor, 2014) which in turn can lead to increased prevalence of AUD in the population.

In terms of socio-demographic correlates, younger age was significantly associated with mental disorders. Epidemiological data across studies, using a range of different methods, has shown higher overall rates of depression over time as well as in successively younger birth cohorts (Wittchen and Uhmann, 2010). A systematic review by (Ferrari *et al*., 2013) identified a ‘time effect’ which suggested that the prevalence of MDD had increased over time. However, the authors acknowledged that other methodological and environmental factors may have resulted in this finding and that further investigation was needed. Other explanations for the higher prevalence include recall bias leading to under-reporting among older adults, lower stigma and better knowledge of mental disorders in the younger cohort leading them to endorse symptoms more openly (Subramaniam *et al*., 2017*a*).

Surprisingly, gender was not strongly associated with MDD in the current study. A few factors could explain the lack of association between gender and MDD in the current study. The last SMHS identified a significant gender difference (Picco *et al*., 2017) which was widely reported by the local media. Experts in mood disorder as well as those with a lived experience of mental illness have elaborated on the tendency of males downplaying the symptoms of depression due to gender norms as well as stigmatization (Addis, 2008). Extensive coverage could have improved awareness in males. Further examination of our findings revealed that while the prevalence of depression in females did not decrease since 2010, there was an increase in lifetime depression among males from 4.3% in SMHS 2010 to 5.6% in SMHS 2016. Van de Velde *et al*. (2010) examined gender differences in depression across 23 countries in Europe (European Social Survey −3) using the Center for Epidemiological Studies Depression Scale. In general women reported significantly higher levels of depression than men, except in Ireland, Finland and Slovakia. Hierarchical linear models found that socioeconomic and family-related factors moderated the relationship between gender and depression. It is thus evident that gender differences in depression vary across countries and both macrosocial and microsocial factors may moderate or mediate the relationship between the two. Future studies need to investigate such contextual factors in further detail in Asian countries to gain a deeper understanding of the gender differences in depression.

Those of Malay ethnicity had lower odds of alcohol abuse and higher odds of OCD as compared to those of Chinese ethnicity. The lower prevalence and risk of alcohol abuse among Malays is readily explained: most Malays in Singapore are Muslims and in Islam, consumption of alcohol is forbidden. However, the higher odds of OCD are difficult to explain and needs further research to elucidate the underlying cause(s).

Educational status was strongly associated with alcohol abuse where those with lower education were more likely to be associated with alcohol abuse. This association has been found in previous studies in the local population (Subramaniam *et al*., 2012*c*; 2017*b*) as well as in other countries (Andrade *et al*., 2002; Park *et al*., 2008). It has been suggested that better-educated people are less likely to engage in risky behaviors, such as smoking and drinking, and are more likely to have healthy behaviors related to diet and exercise. It is also possible that those with lower education adopt unhealthy ways of coping with stress which includes alcohol consumption (Zimmerman and Woolf, 2014). Lastly, education is associated with the wider and more complex issue of socioeconomic status which has a significant influence on alcohol intake, abuse and the general health of populations.

The relationship of unemployment with mental disorders has been established in several studies; unemployment may be a cause or consequence of mental disorders. It is possible that a person with a mental disorder is unable to get or maintain steady employment as a result of the symptoms of the mental disorder. The time that they have to spend on receiving treatment or stigmatization / discrimination on the part of the employers and co-workers (Tsang *et al*., 2007; Nelson and Kim, 2011) might pose difficulties in securing and sustaining full employment. On the other hand, the financial strain of unemployment as well as the psychological and emotional impact of unemployment including the strain on family relationships may also lead to mental illness (OECD, 2008).

The findings of the study must be considered in the light of its limitations. It was not possible to establish the prevalence of all mental disorders due to constraints of time, cost, and respondent burden. In this study, we chose those disorders that are likely to have the greatest impact locally through a comprehensive review of the scientific literature, and in consultation with the policy makers in the Ministry of Health of Singapore. However, this limits comparisons with studies which included all the disorders. It is also possible that we inadvertently excluded a disorder which may be associated with significant burden in this population. This was a household sample so it excluded residents of nursing homes, hospitals and prisons. These groups are likely to have higher rates of mental disorders than the general population, but they also comprise a very small proportion of the total Singapore population. About 30% of the sample was not interviewed – this non-response could lead to underestimation of the true prevalence as mental health determinants differ between responders and non-responders (de Winter *et al*., 2005). Weights were applied to the data to adjust for discrepancies between the sample and population data to account for non-response and sampling variability. Lifetime prevalence estimates are especially subject to recall bias and this may have resulted in lower estimations of the prevalence. Participants may have under-reported or denied their symptoms due to reluctance to share their personal experiences or due to self-stigma. This, however, was minimized in this study by assuring participants that their personal identifiers would not be linked to their individual data and that all results would be presented at the population level and not at the individual level. As this was a cross-sectional study, we are unable to determine incidence of the disorders or the causality and thus are unable to provide explanations for findings that are unique to the population. Lastly, some populations such as those who spoke languages in which the study questionnaires were not available (e.g., Tamil, Chinese dialects) albeit small, would have been excluded from the study thus limiting generalizability to this group.

## Conclusion

The present study showed that mental disorders are prevalent in Singapore with 1 in 7 people in the population having a lifetime mood, anxiety or AUD. The high prevalence of OCD and the increase across the two surveys needs to be tackled at a population level both in terms of creating awareness of the disorder and the need for early treatment. Youth emerge as a vulnerable group who are more likely to be associated with mental disorders and thus targeted interventions in this groups with a focus on youth friendly and accessible care centres may lead to earlier detection and treatment of mental disorders. The results also emphasise the need for prospective longitudinal studies focusing both on a deeper understanding of these disorders and assessing the effectiveness of prevention and treatment efforts.

## Supplementary material

For supplementary material accompanying this paper visit http://dx.doi.org/10.1017/S2045796019000179. click here to view supplementary material

## Availability of data

The data is available on request.

## References

[ref1] Addis ME (2008) Gender and depression in men. Clinical Psychology: Science and Practice 15, 153–168.

[ref2] American Psychiatric Association (1994) Diagnostic and Statistical Manual of Mental Disorders, 4th Edn, (DSM-IV). Washington, DC: Author.

[ref3] Andrade L, Walters EE, Gentil V and Laurenti R (2002) Prevalence of ICD-10 mental disorders in a catchment area in the city of São Paulo, Brazil. Social Psychiatry and Psychiatric Epidemiology 37, 316–325. 12111023 10.1007/s00127-002-0551-x

[ref4] Babor TF (2014) The gathering storm: alcohol abuse among the Chinese in Asia, and the public health response. Malaysian Journal of Chinese Studies 3, 1–20.

[ref5] Bromet E, Andrade LH, Hwang I, Sampson NA, Alonso J, de Girolamo G, De Graaf R, Demyttenaere K, Hu C, Iwata N, Karam AN, Jaur J, Kostyuchenko S, Lepine JP, Levinson D, Matschinger H, Mora ME, Browne MO, Posada-Villa j, Viana MC, Williams DR and Kessler RC (2011) Cross-national epidemiology of DSM-IV major depressive episode. BMC Medicine 9, 90. 21791035 10.1186/1741-7015-9-90 PMC3163615

[ref6] Cheever NA, Rosen LD, Carrier LM and Chavez A (2014) Out of sight is not out of mind: the impact of restricting wireless mobile device use on anxiety levels among low, moderate and high users. Computers in Human Behavior 37, 290–297.

[ref7] Cho MJ, Seong SJ, Park JE, Chung IW, Lee YM, Bae A, Ahn JH, Lee DW, Bae JN, Cho SJ, Park JI, Son J, Chang SM, Hahm BJ, Lee JY, Sohn JH, Kim JS and Hong JP (2015) Prevalence and correlates of DSM-IV mental disorders in South Korean adults: the Korean epidemiologic catchment area study 2011. Psychiatry Investigation 12, 164–170. 25866515 10.4306/pi.2015.12.2.164 PMC4390585

[ref8] Chong SA, Vaingankar J, Abdin E and Subramaniam M (2012) The prevalence and impact of depression among Chinese, Malays and Indians in an Asian multi-racial population. Journal of Affective Disorders 138, 128–136. 22209269 10.1016/j.jad.2011.11.038

[ref9] Corbani IE, Rucci P, Iapichino E, Quartieri Bollani M, Cauli G, Ceruti MR, Gala C and Bassi M (2017) Comparing the prevalence and the risk profile for antenatal depressive symptoms across cultures. The International Journal of Social Psychiatry 63, 622–631. 28805152 10.1177/0020764017725543

[ref10] Demyttenaere K, Bruffaerts R, Posada-Villa J, Gasquet I, Kovess V, Lepine JP, Angermeyer MC, Bernert S, de Girolamo G, Morosini P, Polidori G, Kikkawa T, Kawakami N, Ono Y, Takeshima T, Uda H, Karam EG, Fayyad JA, Karam AN, Mneimneh ZN, Medina-Mora ME, Borges G, Lara C, de Graaf R, Ormel J, Gureje O, Shen Y, Huang Y, Zhang M, Alonso J, Haro JM, Vilagut G, Bromet EJ, Gluzman S, Webb C, Kessler RC, Merikangas KR, Anthony JC, Von Korff MR, Wang PS, Brugha TS, Aguilar-Gaxiola S, Lee S, Heeringa S, Pennell BE, Zaslavsky AM, Ustun TB, Chatterji S and WHO World Mental Health Survey Consortium (2004) Prevalence, severity, and unmet need for treatment of mental disorders in the World Health Organization World Mental Health Surveys. JAMA 291, 2581–2590. 15173149 10.1001/jama.291.21.2581

[ref11] De-Sola Gutiérrez J, Rodríguez de Fonseca F and Rubio G (2016) Cell-phone addiction: a review. Frontiers in Psychiatry 7, 175. 27822187 10.3389/fpsyt.2016.00175 PMC5076301

[ref12] de Winter AF, Oldehinkel AJ, Veenstra R, Brunnekreef JA, Verhulst FC and Ormel J (2005) Evaluation of non-response bias in mental health determinants and outcomes in a large sample of pre-adolescents. European Journal of Epidemiology 20, 173–181. 15792285 10.1007/s10654-004-4948-6

[ref13] Ferrari AJ, Somerville AJ, Baxter AJ, Norman R, Patten SB, Vos T and Whiteford HA (2013) Global variation in the prevalence and incidence of major depressive disorder: a systematic review of the epidemiological literature. Psychological Medicine 43, 471–481. 22831756 10.1017/S0033291712001511

[ref14] Global System for Mobile Communications (GSMA) (2018) The Mobile Connectivity Index, Global Scores 2018. Available online at https://www.gsma.com/mobilefordevelopment/wp-content/uploads/2018/09/GSMA_Mobile-Connectivity-Index-GLOBAL-Fcous.pdf. Last accessed on 21 December 2018.

[ref15] Guo X, Meng Z, Huang G, Fan J, Zhou W, Ling W, Jiang J, Long J and Su L (2016) Meta-analysis of the prevalence of anxiety disorders in mainland China from 2000 to 2015. Scientific Reports 6, 28033. 27306280 10.1038/srep28033 PMC4910078

[ref16] Ishikawa H, Kawakami N and Kessler RC, World Mental Health Japan Survey Collaborators (2016) Lifetime and 12-month prevalence, severity and unmet need for treatment of common mental disorders in Japan: results from the final dataset of World Mental Health Japan Survey. Epidemiology and Psychiatric Sciences 25, 217–229. 26148821 10.1017/S2045796015000566 PMC5144586

[ref17] Ishikawa H, Tachimori H, Takeshima T, Umeda M, Miyamoto K, Shimoda H, Baba T and Kawakami N (2018) Prevalence, treatment, and the correlates of common mental disorders in the mid 2010's in Japan: the results of the World Mental Health Japan 2nd Survey. Journal of the Affective Disorders 241, 554–562. 10.1016/j.jad.2018.08.050 30153639

[ref18] Kessler RC and Ustün TB (2004) The World Mental Health (WMH) survey initiative version of the World Health Organization (WHO) Composite International Diagnostic Interview (CIDI). International Journal of Methods in Psychiatric Research 13, 93–121. 15297906 10.1002/mpr.168 PMC6878592

[ref19] Kessler RC, Demler O, Frank RG, Olfson M, Pincus HA, Walters EE, Wang P, Wells KB and Zaslavsky AM (2005) Prevalence and treatment of mental disorders, 1990 to 2003. The New England Journal of Medicine 352, 2515–2523. 15958807 10.1056/NEJMsa043266 PMC2847367

[ref20] Kessler RC, Avenevoli S, Costello EJ, Georgiades K, Green JG, Gruber MJ, He JP, Koretz D, McLaughlin KA, Petukhova M, Sampson NA, Zaslavsky AM and Merikangas KR (2012) Prevalence, persistence and sociodemographic correlates of DSM-IV disorders in the National Comorbidity Survey Replication Adolescent Supplement. Archives of General Psychiatry 69, 372–380. 22147808 10.1001/archgenpsychiatry.2011.160 PMC3445020

[ref21] Liao SC, Chen WJ, Lee MB, Lung FW, Lai TJ, Liu CY, Lin CY, Yang MJ and Chen CC (2012) Low prevalence of major depressive disorder in Taiwanese adults: possible explanations and implications. Psychological Medicine 42, 1227–1237. 22051196 10.1017/S0033291711002364

[ref22] Nelson RE and Kim J (2011) The impact of mental illness on the risk of employment termination. The Journal of Mental Health Policy and Economics 14, 39–52. 21642748

[ref23] Organisation for Economic Co-operation and Development (OECD) (2008) Are all jobs good for your health? The impact of work status and working conditions on mental health. *OECD Employment Outlook*. Chapter 4.

[ref24] Park JT, Kim BG and Jhun HJ (2008) Alcohol consumption and the CAGE questionnaire in Korean adults: results from the Second Korea National Health and Nutrition Examination Survey. Journal of Korean Medical Science 23, 199–206. 18437000 10.3346/jkms.2008.23.2.199 PMC2526429

[ref25] Patten SB, Williams JV, Lavorato DH, Wang JL, McDonald K and Bulloch AG (2016) Major depression in Canada: what has changed over the past 10 years? Canadian Journal of Psychiatry 61, 80–85. 27253698 10.1177/0706743715625940 PMC4784240

[ref26] Picco L, Subramaniam M, Abdin E, Vaingankar JA and Chong SA (2017) Gender differences in major depressive disorder: findings from the Singapore Mental Health Study. Singapore Medical Journal 58, 649–655. 27526704 10.11622/smedj.2016144 PMC5691228

[ref27] Ryder AG, Yang J, Zhu X, Yao S, Yi J, Heine SJ and Bagby RM (2008) The cultural shaping of depression: somatic symptoms in China, psychological symptoms in North America? Journal of Abnormal Psychology 117, 300–313. 18489206 10.1037/0021-843X.117.2.300

[ref28] Shen YC, Zhang MY, Huang YQ, He YL, Liu ZR, Cheng H, Tsang A, Lee S and Kessler RC (2006) Twelve-month prevalence, severity, and unmet need for treatment of mental disorders in metropolitan China. Psychological Medicine 36, 257–267. 16332281 10.1017/S0033291705006367

[ref29] Soreni N, Streiner D, McCabe R, Bullard C, Swinson R, Greco A, Pires P and Szatmari P (2014) Dimensions of perfectionism in children and adolescents with obsessive-compulsive disorder. Journal of the Canadian Academy of Child Adolescent Psychiatry 23, 136–143. 24872829 PMC4032082

[ref30] Steel Z, Marnane C, Iranpour C, Chey T, Jackson JW, Patel V and Silove D (2014) The global prevalence of common mental disorders: a systematic review and meta-analysis 1980–2013. International Journal of Epidemiology 43, 476–493. 24648481 10.1093/ije/dyu038 PMC3997379

[ref31] Subramaniam M, Vaingankar J, Heng D, Kwok KW, Lim YW, Yap M and Chong SA (2012 *a*) The Singapore Mental Health Study: an overview of the methodology. International Journal of Methods Psychiatric Research 21, 149–157. 10.1002/mpr.1351 PMC6878512 22331628

[ref32] Subramaniam M, Abdin E, Vaingankar JA and Chong SA (2012 *b*) Obsessive--compulsive disorder: prevalence, correlates, help-seeking and quality of life in a multiracial Asian population. Social Psychiatry and Psychiatric Epidemiology 47, 2035–2043. 22526825 10.1007/s00127-012-0507-8

[ref33] Subramaniam M, Abdin E, Vaingankar J, Phua AM, Tee J and Chong SA (2012 *c*) Prevalence and correlates of alcohol use disorders in the Singapore Mental Health Survey. Addiction 107, 1443–1452. 22296228 10.1111/j.1360-0443.2012.03830.x

[ref34] Subramaniam M, Abdin E, Picco L, Pang S, Shafie S, Vaingankar JA, Kwok KW, Verma K and Chong SA (2017 *a*) Stigma towards people with mental disorders and its components – a perspective from multi-ethnic Singapore. Epidemiology and Psychiatric Sciences 26, 371–382. 27018715 10.1017/S2045796016000159 PMC5647661

[ref35] Subramaniam M, Mahesh MV, Peh CX, Tan J, Fauziana R, Satghare P, Gupta B, Gomathinayagam K and Chong SA (2017 *b*) Hazardous alcohol use among patients with schizophrenia and depression. Alcohol 65, 63–69. 29084631 10.1016/j.alcohol.2017.07.008

[ref36] Tang YL, Xiang XJ, Wang XY, Cubells JF, Babor TF and Hao W (2013) Alcohol and alcohol-related harm in China: policy changes needed. Bulletin of the World Health Organization 91, 270–276. 23599550 10.2471/BLT.12.107318 PMC3629448

[ref37] Tsang HW, Angell B, Corrigan PW, Lee YT, Shi K, Lam CS, Jin S and Fung KM (2007) A cross-cultural study of employers’ concerns about hiring people with psychotic disorder: implications for recovery. Social Psychiatry Psychiatric Epidemiology 42, 723–733. 17522747 10.1007/s00127-007-0208-x

[ref38] Van de Velde S, Bracke P and Levecque K (2010) Gender differences in depression in 23 European countries: Cross-national variation in the gender gap in depression. Social Science & Medicine 71, 305–313. 20483518 10.1016/j.socscimed.2010.03.035

[ref39] Wittchen HU and Uhmann S (2010) The timing of depression: an epidemiological perspective. Medicographia 32, 115–126. (https://www.medicographia.com/wp-content/pdf/Medicographia103.pdf).

[ref40] World Health Organization (1992) The ICD-10 Classification of Mental and Behavioural Disorders: Clinical Descriptions and Diagnostic Guidelines. Geneva: World Health Organization.

[ref41] World Health Organization (2017) Depression and Other Common Mental Disorders: Global Health Estimates. Geneva: World Health Organization. Licence: CC BY-NC-SA 3.0 IGO. Available online at http://apps.who.int/iris/bitstream/handle/10665/254610/WHO-MSD-MER-2017.2-eng.pdf;jsessionid=FF5A7B1D7D63B6455DEA1C5E6E7332B9?sequence=1. Last accessed on 20 Dec 2018.

[ref42] World Health Organization, Mental Health and Substance Abuse Unit (2013) Reducing harm from alcohol use: good practices. (apps.who.int/iris/bitstream/10665/205737/1/b4955.pdf). Accessed 17 August 2018.

[ref43] Wu KD and Cortesi GT (2009) Relations between perfectionism and obsessive-compulsive symptoms: examination of specificity among the dimensions. Journal of Anxiety Disorder 23, 393–400. 10.1016/j.janxdis.2008.11.006 19110399

[ref44] Zimmerman E and Woolf S (2014) Understanding the Relationship Between Education and Health (Discussion Paper). Washington, DC: Institute of Medicine. (https://nam.edu/wp-content/uploads/2015/06/BPH-UnderstandingTheRelationship1.pdf). Accessed 14 August 2018.

