# Supplementary material for: Tracking the mental health of a nation: prevalence and correlates of mental disorders in the second Singapore mental health study
Source: Epidemiol Psychiatr Sci. 2019 Apr 5;29:e29. doi: 10.1017/S2045796019000179 (PMC8061188; doi:10.1017/S2045796019000179)
Supplement: Supplementary file 1 [file S2045796019000179sup001.docx]

**Supplementary Table 1: Socio-demographic distribution of the sample (N = 6,126)**

| **Sociodemographic characteristics** | | **N** | **Unweighted**  **%** | **Weighted**  **%** |
| --- | --- | --- | --- | --- |
| **Age Group (years** | 18-34 | 1707 | 27.9 | 30.4 |
| **(Mean = 45.2)** | 35-49 | 1496 | 24.4 | 29.6 |
|  | 50-64 | 1626 | 26.5 | 26.9 |
|  | 65+ | 1297 | 21.2 | 13.1 |
| **Gender** | Female | 3058 | 49.9 | 50.4 |
|  | Male | 3068 | 50.1 | 49.6 |
| **Ethnicity** | Chinese | 1782 | 29.1 | 75.7 |
|  | Malay | 1990 | 32.5 | 12.5 |
|  | Indian | 1844 | 30.1 | 8.7 |
|  | Others | 510 | 8.3 | 3.1 |
| **Marital Status** | Never Married | 1544 | 25.2 | 31.0 |
|  | Married | 3843 | 62.7 | 59.8 |
|  | Divorced / Separated | 343 | 5.6 | 5.2 |
|  | Widowed | 396 | 6.5 | 4.1 |
| **Education** | Primary and below | 1187 | 19.4 | 16.3 |
|  | Secondary | 1648 | 26.9 | 23.0 |
|  | Pre-U/Junior College | 304 | 5.0 | 6.0 |
|  | Vocational/ITE | 508 | 8.3 | 6.3 |
|  | Diploma | 1024 | 16.7 | 19.0 |
|  | University | 1455 | 23.8 | 29.4 |
| **Employment** | Employed | 4055 | 66.2 | 72.0 |
|  | Economically inactive* | 1716 | 28.0 | 22.7 |
|  | Unemployed | 354 | 5.8 | 5.3 |
| **Household Income (SGD/ month)** | Below 2000 | 1147 | 21.0 | 16.5 |
|  | 2,000 – 3,999 | 1331 | 24.4 | 20.0 |
|  | 4,000 – 5,999 | 1113 | 20.4 | 21.4 |
|  | 6,000 – 9,999 | 1003 | 18.4 | 21.8 |
|  | 10,000 & above | 861 | 15.8 | 20.3 |

* Includes homemakers, students and retirees / pensioners; SGD- Singapore Dollars
